# Plastic debris dataset on the Seine riverbanks: up to 38,000 pre-production plastic pellets reported per square meter

**DOI:** 10.1016/j.dib.2025.111735

**Published:** 2025-05-31

**Authors:** Romain Tramoy, Laurent Colasse, Johnny Gasperi, Bruno Tassin

**Affiliations:** a LEESU*,* Univ Paris Est Créteil*,* Ecole Des Ponts*,* Creteil*,* France; b Université de Rouen Normandie*,* Polymères Biopolymères Surfaces (PBS) UMR 6270 CNRS*,* 76000 Rouen*,* France; c Univ Gustave Eiffel*,* GERS-LEE*,* 44344 Bouguenais*,* France; d Institut de Recherche en Sciences et Techniques de la Ville*,* IRSTV*,* CNRS, 1 Rue de La Noë*,* 44321 Nantes*,* France; e LEESU, Ecole Des Ponts*,* Univ Paris Est Creteil*,* Marne‑La‑Vallée*,* France

**Keywords:** Plastic pollution, Estuary, River, Soil, Microplastics, Nurdle

## Abstract

Plastic pollution in rivers is a major source for plastic pollution into the ocean. However, it is now recognized that plastics may accumulate in rivers for years, especially in estuaries, before reaching the ocean. This long residence time favours fragmentation of macroplastics into smaller and smaller pieces, but relative data are still carse. Here we present data from the downstream part of the Seine estuary in a historical deposit full of plastic debris, with the highest concentration of industrial plastic pellet ever reported in France. Plastic debris (down to 6 mm according to sieving limits) were classified using the updated European classification J-list. The sampled site is located close to the river mouth within a national natural reserve, surrounded by international harbour activities and two major industrial plastic producers: Total Energy and Exxon Mobil. A surface of only 1 m^2^ was sampled in a visual maximum of plastic pollution. Over 100,000 plastic debris were counted or estimated when it comes to plastic debris <6 mm. Items were classified and weighted by category for a total mass higher than 4 kg. By mass (count), 24 % (38 %) of total plastic debris were pre-production plastic pellets, 21 % (1 %) were unidentified, colourful, plastic fragments (*≥* 2.5 cm), and 19 % (33 %) were unidentified, colourful, plastic fragments (<2.5 cm).

Specifications Table

**Table:** 

Subject	*Pollution*
Specific subject area	*Plastic pollution in rivers. pre-Production Plastic Pellets.*
Type of data	Table, Figure. Raw, Analyzed data
Data collection	*One square meter was sampled in a maximum visual pollution site. All debris were collected with a metal trowel to the point of scratching the soil surface. They were then transported and stored in buckets. Visual identification following European classifications (J-code, G-code and OSPAR) was then performed in the lab after sieving all material in three size classes: <6mm, 6–25mm and*≥*25mm.*
Data source location	*Sampling performed at the following GPS coordinates:* *49.46452, 0.43911*
Data accessibility	Repository name: Pangaea.com Data identification number: PANGAEA.974719719 Direct URL to data: 10.1594/PANGAEA.974719

## 1 Value of the Data

• Identified plastic items in riverbanks according to European classifications of litter (J-code, G-code and OSPAR).
• Reporting items in number, mass and volume for conversions between units in other studies dealing with plastic litter in rivers.
• Amount of pre-production plastic pellets per square meter is reported at levels never reported before.
• Baseline amount for banned items like plastic cotton bud sticks (banned from EU in 2020) or non-attached caps for drinks (banned from EU in 2024).
• Target new sites for large scale cleaning.

## 2 Background

There is still little ground-truth data related to visible plastic pollution in long-term accumulation zones in rivers, especially in industrialized estuaries like the Seine estuary. In Tancarville area, very high levels of pre-production plastic pellets are found, and this site is only the second one investigated with a complete characterization of all visible plastic debris [1]. Since mitigation policies are under discussion at the European level to tackle pellets pollution (https://oeil.secure.europarl.europa.eu/oeil/popups/ficheprocedure.do?reference=2023/0373(COD)&l=en), all available relative data are welcome to focus also on production sites and their surroundings, and not only on maritime transportation routes as a source of pellets.

## 3 Data Description

In this report, an inventory of plastic items is presented (cf. csv file), coming from a national natural reserve along the Seine River. Data are representative of the long-term plastic pollution occurring in this river with a huge accumulation of plastic debris, however difficult to date.

More than 100,000 plastic debris were counted (plastic debris *≥* 6 mm) or estimated (plastic debris <6 mm, including plastic pellets), classified and weighted by category using international classification commonly used for marine litter. It represents more than 4 kg of plastic per square meter.

**Fig. 1:**
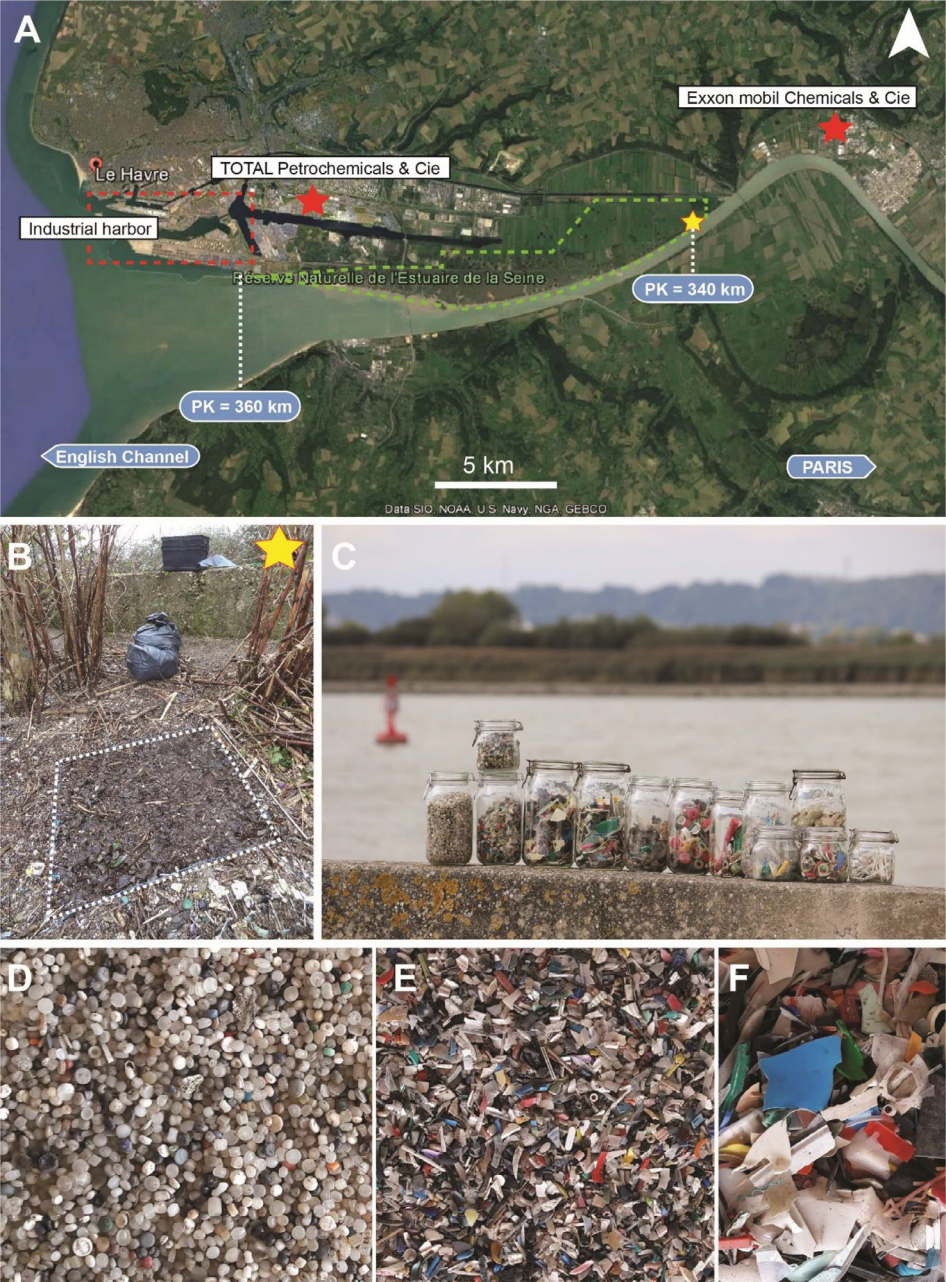
A, map of the Seine estuary with the sampling site (yellow star) and plastic producers (red stars). Pk for kilometric point, which is the distance from the river source. B, the sampled square meter. C, plastic debris collected and sorted in glass jars. D, plastic pre-production plastic pellets. E, unidentified fragments <2.5 cm. F, unidentified fragments *≥* 2.5 cm.

In the dataset, categories of items were ordered by mass, while empty categories (with zero occurrence) were removed from the list for more clarity. By mass, the Top 3 categories of items are pre-production plastic pellets (24 %), unidentified, colourful, plastic fragments *≥* 2.5 cm (21 %), and fragments <2.5 cm (19 %). They represent 64 % of the total count. By count, the Top 3 categories of items are pre-production plastic pellets (38 %), unidentified, colourful, plastic fragments <2.5 cm (33 %), and fragments of foamed polystyrene <2.5 cm (22 %). They represent 93 % of the total count. Plastic industries (production and transport of plastic pellets) implemented in the Seine estuary (Fig. 1) are revealed as a major contributor of the local plastic pollution, while fragmentation of other plastic items is most likely occurring on a large spatiotemporal scale in the entire estuary.

Other items of concerns are featuring in the TOP 10 by mass like pyroplastics (TOP 4) from unknown sources, plastic caps/lids from drinks and rings (TOP 5 and 10 respectively), plastic toys (TOP 6) or plastic cotton bud sticks (Top 7). Notice that plastic caps must be attached to their bottles since 2024 and that plastic cotton bud sticks are banned from European market since 2020. Those items in the future will then progressively become markers of historical deposits.

Data are presented in the external repository with the following column labels (see csv file):

- Sample ID (Type Code): hierarchical system using series of letter separated by “_” with, from the left to the right, level 1 for Material, level 2 for use/economic sector, level 3 to 5 for further detail on item type and size class. See Fleet et al. (2021) for more details.
- Sample ID (J-Code classification), (G-code classification) and (OSPAR classification): the three classifications used with the J-list (J-Code) gathering G-code and OSPAR.
- Sample comment: items and description according to their J-Code.
- “Microplastic” <6 mm: number of items <6 mm. Upper size limit for microplastic is normally 5 mm. For this study, we used a sieve of 6 mm to better separate pre-Production Plastic Pellets. “Microplastics” estimates were based on extrapolated visual counting of several subsamples in the 6 mm sieved fraction.
- “Mesoplastic” 6–25 mm: number of items between 6 and 25 mm. Notice the regular range for mesoplastic is 5–25 mm.
- Macroplastic: number of items *≥* 25 mm
- Plastic tot [#]: “Microplastic” + “Mesoplastic” + Macroplastic
- Perc [%]: fraction of the category either in numb or in mass relative to the total items.
- Mass [g]: total mass of “micro”, “meso” and macro items of the same category in g
- Vol [ml]: Volume estimated of the category in mL.
- Comment: additional information about items or count estimates for smallest items

## 4 Experimental Design, Materials and Methods

### 4.1 Site description

Plastic litter was collected in February 2020 in the downstream part of the Seine River estuary close to Tancarville, 90 km downstream of Rouen and 20 km upstream the river mouth (Fig. 1A; GPS coordinates: 49.46452, 0.43911). The Seine estuary is a macrotidal estuary with a tidal range up to 7 m. The sampled site is located within a national natural reserve and between two main plastic producers: Total and Exxon Mobil. The depositional environment is a dried marsh regularly flooded in winter by the river just below an ancient towpath.

### 4.2 Sampling method

Plastic litter was exhaustively collected by hands and with a metal trowel in a quadrat of 1 m^2^ in a visual maximum of plastic accumulation until the soil was reached (Fig. 1B). Samples were stored in plastic bags of 50 L. They were dried at 50 °C in the oven for several days, before analysis.

### 4.3 Analysis and classification

All plastic debris above 5 mm were sorted and counted one by one in the lab, classified by size and category, and weighted (Fig. 1 C-F). Plastic items were classified according to the updated European MSFD TG ML^1^ and OSPAR^2^ classifications, joined as the J-list [2]. Size classes were determined based on the largest dimension for each piece. Pellets were estimated based on their total mass divided by the average mass of one pellet (0,025 g). Other microplastics estimates were based on extrapolated visual counting of several subsamples in the 6 mm sieved fraction. Hence, results are less confident than for pellets and above all than macroplastics count.

## Limitations

The main limitation relies on the estimation of plastics <6 mm, except for pellets easily recognizable. For the other micro fragments, the lower detection limit is the limit imposed by the naked eye typically slightly lower than 1 mm. Thus, smaller microplastics are not accounted for in this dataset and the estimation of larger microplastics are based on rough estimates. In addition, they were sieved at 6 mm for more convenience regarding pellets (typically around 3–5 mm) and not at 5 mm, which is slightly different from the regular separation between macro- and microplastics.

A minor limitation relies on the representativeness of the sample, because the sample was collected in maximum visual pollution. So, it represents a worse case.

## Ethics Statement

The authors have read and follow the ethical requirements for publication in Data in Brief and confirm that the current work does not involve human subjects, animal experiments, or any data collected from social media platforms.

## CRediT Author Statement

**Laurent Colasse:** Conceptualization, Methodology, Investigation. **Romain Tramoy:** Validation, Data curation, Visualization, Writing Original Draft. **Johnny Gasperi:** Supervision, Writing – Review & Editing, Project administration. **Bruno Tassin:** Supervision, Writing – Review & Editing, Project administration.

## Data Availability

PANGAEAPlastic debris dataset on the Seine riverbanks: up to 38 000 (1 kg) pre-production plastic pellets reported per square meter. (Original data).

## Footnotes

1 Marine Strategy Framework Directive Technical Group on Marine Litter.

2 Oslo Paris commission on protecting and conserving the North-East Atlantic.
